# Intention Prediction and Human Health Condition Detection in Reaching Tasks with Machine Learning Techniques †

**DOI:** 10.3390/s21165253

**Published:** 2021-08-04

**Authors:** Federica Ragni, Leonardo Archetti, Agnès Roby-Brami, Cinzia Amici, Ludovic Saint-Bauzel

**Affiliations:** 1 Department of Mechanical and Industrial Engineering, University of Brescia, via Branze, 38, 25123 Brescia, Italy; f.ragni@unibs.it (F.R.); l.archetti012@studenti.unibs.it (L.A.); 2 ISIR (Institute of Intelligent Systems and Robotics), UMR CNRS 7222, AGATHE Team INSERM U 1150, Sorbonne Université, 75005 Paris, France; roby-brami@isir.upmc.fr (A.R.-B.); ludovic.saint-bauzel@sorbonne-universite.fr (L.S.-B.)

**Keywords:** human intention prediction, wearable sensors, machine learning, reaching movement

## Abstract

Detecting human motion and predicting human intentions by analyzing body signals are challenging but fundamental steps for the implementation of applications presenting human–robot interaction in different contexts, such as robotic rehabilitation in clinical environments, or collaborative robots in industrial fields. Machine learning techniques (MLT) can face the limit of small data amounts, typical of this kind of applications. This paper studies the illustrative case of the reaching movement in 10 healthy subjects and 21 post-stroke patients, comparing the performance of linear discriminant analysis (LDA) and random forest (RF) in: (i) predicting the subject’s intention of moving towards a specific direction among a set of possible choices, (ii) detecting if the subject is moving according to a healthy or pathological pattern, and in the case of discriminating the damage location (left or right hemisphere). Data were captured with wearable electromagnetic sensors, and a sub-section of the acquired signals was required for the analyses. The possibility of detecting with which arm (left or right hand) the motion was performed, and the sensitivity of the MLT to variations in the length of the signal sub-section were also evaluated. LDA and RF prediction accuracies were compared: Accuracy improves when only healthy subjects or longer signals portions are considered up to 11% and at least 10%, respectively. RF reveals better estimation performance both as intention predictor (on average 59.91% versus the 62.19% of LDA), and health condition detector (over 90% in all the tests).

## 1. Introduction

Detecting and predicting human intentions by collecting and analyzing body signals are among the main goals in human–robot interaction [1]. These challenging tasks and their relevance in daily-living applications are gaining importance, for instance due to the spreading of collaborative robots (cobots) for human–robot cooperation. An accurate and real-time interpretation of the motion intention could ease the achieving of effective human–machine coordination strategies [2] for interactive robotic interfaces or diagnostic systems [3].

For a non-invasive detection of body signals several kinds of sensors can be adopted, such as accelerometers [4], electroencephalography (EEG) [3], or surface electromyography (sEMG) [2,5,6]. In the last years, investigations on the suitability of wearable sensors for the pattern recognition of human movements have been widely conducted [2,3,7], also evaluating the effect of the sensors positioning on the acquired data [8,9]. Wearable sensors assure non-invasive analyses and are compliant to full integration with pre-existing systems or commercially available devices [1]. In addition, they allow assessing body signals or motion properties (such as acceleration and velocity) to reconstruct an observed movement [10], overcoming potential inter- and intra-subjects anatomical variability that could affect the measurement quality [11].

Since movements are subject-dependent, and body signals are sensitive to lack of repeatability [2], the complexity of the human intention prediction task even increases in specific scenarios, such as the clinical environment, where the pathological subject can behave according to peculiar or unpredictable motion patterns. Within this context, laboratory-based optical systems for the movement analysis are widely adopted for periodical monitoring and assessment of the stroke condition during rehabilitation [12], since they enable the measurement of multiple bio-signals, recognized as useful in both detecting pathological symptoms and improving the rehabilitation healing rate [13]. A thorough knowledge of the expected natural behavior and motion patterns in the healthy subject becomes therefore fundamental to perform a correct assessment of the subject condition.

Among all the possible movements affecting the activities of daily living, the reaching task undoubtedly plays a crucial role [14], given the importance of its functional aim [15].

Since a defined movement can be performed according to many different strategies [7,16], the use of predictive models and machine learning algorithms is particularly suitable to analyze the signals with the purpose of predicting the human movement intention [2]. Developing effective working methodologies for the processing of body signals becomes therefore necessary, and machine learning techniques (MLT) can face the limit of small data amounts, typical of this kind of applications.

Literature provides several examples of MLT applied to human movement analysis; for instance, in 2014 Romaszewski et al. applied Linear Discriminant Analysis (LDA), Support Vector Machine and k Nearest Neighbor algorithms to identify natural hand gestures [17]. In 2015, Li et al. discriminated eight different movements of the upper limb exploiting the Random Forest (RF) algorithm for the analysis of optoelectronic data [18], whereas in 2020, Robertson et al. applied the quadratic discriminant analysis to data acquired with a Kinect camera (© Microsoft Corporation, Redmond, WA, USA) to discriminate between healthy subjects and patients affected by Cerebellar Ataxia [19].

Furthermore, deep learning (DL) techniques have been applied to the study of gait data through recurrent neural networks (RNN), deep neural networks (DNN), or dedicated DL approaches [20]. In particular, after detecting the motion through electromyography signals [21] or wearable sensors [22,23], DL techniques have been applied to analyze peculiar movement features. In 2016, Illias et al. [24] also applied the NN model to discriminate the gait patterns between healthy children and children affected by autism. In the human–robot interaction (HRI) field, Liu et al. [25] have applied DL techniques combined with data acquired through 3D body skeletons, 2D RGB images, and optical flows, to identify humans’ intention and build a framework of human–robot interaction. A similar application was also developed in 2019 by Li et al. [26]. In the clinical field, DL techniques have also been applied to detect and study hand movement pattern and its changes, and to determine possible disease. Several researchers have measured this phenomenon using computer vision techniques, such as gesture recognition, analyzing the signal of optical markers placed on the hand joints [27]. Other researchers apply DL algorithms to measure hand parameters detected with a vision system [28,29].

Focusing on the analysis of the reaching movement in both healthy subjects and post-stroke patients, this study aimed to compare the performance of LDA and RF MLT in: (i) predicting subjects intention of moving towards a target or a specific direction (*intention prediction*), and (ii) detecting if the subject is behaving according to a healthy or a pathological pattern, and if the possible damage affected the right of left hemisphere (*health condition detection*). Analyzed data were captured with wearable electromagnetic sensors, and only a first section of the acquired signals was exploited for the prediction and detection processes. Further analyses investigate the possibility of detecting with which arm (left or right hand) the motion is performed by the subject, and the sensitivity of the evaluated MLT to variations in the length of the evaluated signal section.

Compared to previous works in scientific literature, this paper presents novelty aspects in the methodological approach. In particular:

(i). The same dataset is exploited performing different set of analyses, to assess suitability and performance of two MLT with respect to different purposes;
(ii). The performance of LDA and RF are compared for the specific analysis of the reaching movement;
(iii). With particular reference to the previous work of Archetti et al. [30], the analysis of a dataset which also includes data collected from post-stroke patients on one side allowed a more robust evaluation of the performance of LDA and RF as intention predictors, and on the other side enabled the implementation of a new level of analysis, evaluating the performance of LDA and RF as health condition detectors.

## 2. Materials and Methods

An experimental campaign was designed, and the study was approved by CPP Ile de France 8 ethical committee of Hôpital Am-449 broise Paré (ID RCB 2009–A00028-49, 19 June 2009). The study was conducted in accordance with the guidelines of the 448 Declaration of Helsinki.

### 2.1. Participants

For the experimental campaign, a convenience sample of 31 subjects was recruited: ten healthy subjects (6 females; mean age: 51 years, range [29;71] years; 1 left-handed) as control group, and 21 patients who experienced a first ischemic or hemorrhagic stroke with cortical and/or subcortical lesions. Among them, three subjects were left-handed and 18 right-handed (9 females; mean age: 48 years, range [20;71] years).

For the pathological subjects, tests were performed at least 3 months post botulinum toxin injection to assure the absence of lingering effects of the toxin on the human body. Exclusion criteria were: (i) patients with shoulder pain, (ii) previous shoulder pathologies, (iii) multiple or bilateral cerebral lesions, (iv) acute algoneurodystrophy, (v) cerebellar involvement or comprehension deficit, and (vi) range of motion of the upper limbs that does not allow the reaching movement. Within the subset of pathological subjects, ten patients presented a right hemisphere damage (RHD), and the remaining 11 a left hemisphere damage (LHD).

Inclusion criteria for the control group were: (i) age over 18 years old, (ii) no previous or current orthopedic or neurological pathology of the upper arm.

### 2.2. Acquisition Protocol

All testing sessions were performed in the same environmental conditions, i.e., during the morning and in the controlled environment of the Laboratoire de Neurophysique et Physiologie at the Hôpital R. Poincaré, Garches (France). During each session, at first a preliminary trial was carried out to familiarize the subject with the procedure, then the subject was asked to perform six repetitions of unilateral sitting reaching movement: three cycles with the left arm, and three with the right arm. As described in Robertson et al. [19] and Archetti et al. [30], the initial condition consisted in the subject with the hand placed on a red cross placed on the table plane in line with the shoulder, the forearm in mid-prone, the elbow flexed to 90°, and the humerus positioned along the vertical direction. In each repetition, the subject was asked to touch the target identified by the operator, among a set of possible pre-defined positions, which depicts combinations of the three directions left, center, and right, of the two quotes high and low, and of the two distances proximal and distal. Although the subject was oblivious to it, the target sequence submitted by the operator was standardized: close-middle (CM), far-internal (FI), high-external (HE), far-middle (FM), close-external (CE), high-internal (HI), close-internal (CI), far-external (FE), and high-middle (HM). The subjects were instructed to touch the target with a provided pointer and return to the starting position, performing the movement with opened eyes. No instructions were given on accuracy and speed, other than touching the target at a comfortable speed.

### 2.3. Experimental Setup

The subject was seated on a chair adjusted in height to make the table surface at the navel level. A wide strap assured the subject’s trunk to be fixed to the chairback.

A trained operator instrumented the subject with a wrist splint, provided with a pointer to simulate an extended index finger, and with four electromagnetic sensors. The sensors were located on (i) acromion, (ii) upper third of humerus, (iii) wrist dorsum, and (iv) manubrium. During the acquisitions, an electromagnetic tracking system (Polhemus Space Fastrak, Colchester, VT, USA) was used: The system provides the position and orientation of each sensor as timestamped vector triplets (X, Y, Z) and (α, β, γ) respectively, at a frequency of 30 Hz. The assured root mean square (RMS) static accuracy and resolution of the system are respectively 0.8 mm and 0.0005 [cm/cm of range] for the position receivers, and 0.15° and 0.025° for the orientation receiver.

**Figure 1:**
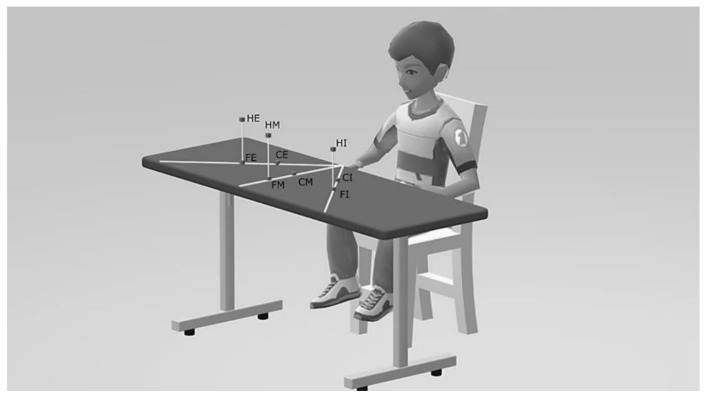
Schematic of the experimental setup. Target positions are indicated as: close-middle (CM), far-internal (FI), high-external (HE), far-middle (FM), close-external (CE), high-internal (HI), close-internal (CI), far-external (FE), and high-middle (HM).

As schematically depicted in Figure 1, nine targets were located within three planes, each of them orthogonal to the table surface and passing through one of the following directions: the parasagittal straight line emanating from the subject’s shoulder for the middle direction, and the straight lines positively and negatively inclined at 45° with respect to the middle plane, as internal and external directions, respectively.

The distance between target and subject was defined with respect to the length of the equivalent anatomical upper limb, meant as the distance between the position of the sensor located at the acromion and the end of the pointer. Two distances were evaluated: (a) close, corresponding to 65% of the total upper limb length, and (b) far, equal to 90% of the upper limb length. Considering the quote parameter, the six targets in low configuration were placed at 70 mm of height from the table level, whereas the three targets in high configuration were located above the corresponding distal targets, at the same quote of the acromion from the table surface.

### 2.4. Data Treatment

Linear position and orientation provided by each sensor were imported and elaborated in MATLAB (© The MathWorks, Inc., Natick, MA, USA) environment. Data processing was performed using an Intel^®^ (© Intel Corporation, Santa Clara, CA, USA) Core™ i7-8565U processor (1.80 GHz) on a machine running a Windows 10 Home (© Microsoft Corporation, Redmond, WA, USA) operative system.

The acquired signals were initially trimmed to the actual motion section to define a dataset of coherent data, comparable among subjects and among trials. To detect starting and ending points of the reaching movement, the absolute value of the velocity of the hand was analyzed. The absolute value of the hand position was computed as the vectorial composition of the signal components along the three directions X, Y, and Z, and the hand velocity was numerically evaluated according to a custom two-point derivative approximation [30]. This signal was then filtered to remove noise with a fourth-order zero-phase low-pass Butterworth filter, according to literature indications [31,32]. For the filter, a cut-off frequency of 3 Hz was adopted [33]. The subject resting condition was defined as the mean value of the first and last ten acquired data samples, each of them corresponding to a time interval of 0.33 s. Starting and ending points of the reaching movement were automatically detected by a custom-developed code, as the first and last time instant, respectively, in which the absolute value of the position first derivative exceeds an imposed threshold. This threshold, initially set to 9 × 10^−3^ mm/s, was iteratively reduced by 1 × 10^−3^ mm/s, and the estimation of starting and ending point was updated, until the variance of the velocity signal from the beginning to the identified starting point, and from the identified ending point on, were below the threshold itself.

To normalize data in amplitude, anthropometric quantities were computed for each subject from the positions of the sensors on hand, arm, and shoulder. In each trial, the relative distances hand-to-arm, arm-to-shoulder, and shoulder-to-trunk of the sensors were calculated during the subject resting phase. The average values of these nine quantities for each subject were then computed and adopted as reference values for the normalization process.

Finally, the second derivative of sensors position was also numerically computed applying the custom two-point derivative approximation, to simulate data coming from fictitious accelerometers placed on subjects. The resulting signal was then filtered applying the same low-pass Butterworth filter previously described (fourth-order zero-phase, cut-off frequency of 3 Hz).

To identify a feature set for the implementation of the machine learning algorithms four signals were considered: (i) the linear position computed by the sensors position (SP) components, (ii) the modulus of the sensors velocity, i.e., the first derivative of SP, (iii) the modulus of the sensors acceleration, i.e., the second derivative of SP, and (iv) the angular position computed by the measured Euler angles. For the purpose of the features extraction, only a section of the overall signal was analyzed as the observation window (OW). Two different approaches were adopted for the evaluation of the OW size: (i) a subject- and trial-dependent strategy, based on a *custom window* which computes the observation time using the information on the motion length of the specific trial, and (ii) a generalized approach, based on an *average window* which exploits the dataset of all the available data, from all the subjects and trials, to compute a fixed OW.

For the implementation of the machine learning algorithms, the minimum, maximum and root mean square values of linear and angular position, velocity and acceleration were evaluated as features from the source signals. For each subject and trial, all the computed features were rescaled to −0.80, +0.80. The LDA and RF algorithms were applied for the data treatment, and only data from the sensors placed on hand and arm were used in both the analyses, since a first set of preliminary evaluations suggested that the data gathered from the other sensors provided negligible contributes. For both the purposes of intention prediction and health condition detection, the algorithms were trained using a subset of randomly selected data. According to the results of a preliminary analysis, the size of those subsets was selected at 85% and 90% of the analyzed dataset, respectively; those thresholds in fact proved to allow a reasonable compromise between computation time and prediction performance according to the expectations of the current purpose. The remaining data were then exploited for the testing phase. For both the algorithms, training time and prediction time were also computed.

#### 2.4.1. Intention Prediction

To evaluate the intention prediction, twenty tests were designed, combining different configurations of data setup parameters, used features, and outputs. As setup parameters, OW evaluation strategies and lengths were considered. For the features, four conditions were evaluated, corresponding to features extracted by different source signals: (i) sensor position (P) and velocity (V), (ii) sensor position, velocity, and Euler angles (E), (iii) sensor position, and iv) sensor acceleration (A). Table 1 synthesizes the test conditions for each test. Considering the OW evaluation strategy, the first ten tests applied the average window method, and the remaining the custom window approach. Focusing on the OW length, two different cases were evaluated: a size equal to 1/7 and 1/10 of the total time extent of the actual motion section. For all the tests, the main output was the expected position of the target that the subject wants to reach; for 16 tests, additional output was the left (L) and right (R) distinction, meant as with which limb the motion was expected to be performed.

Tests were performed on the complete dataset of all the subjects, and compared with the results provided by the analysis of the subsets of healthy and pathological subjects only. For the comparison, the prediction accuracy of the LDA algorithm was calculated as depicted by Nuzzi et al. [34,35]; for the assessment of the RF accuracy, the out-of-bag (OOB) method was adopted. The results were averaged on 200 consecutive tests.

#### 2.4.2. Health Condition Detection

To detect the health condition of the subject, eight tests were designed investigating different combinations of data setup parameters, included features, and outputs. The average window approach was adopted as OW evaluation strategy for all the tests. Two combinations of source signals were considered for the features evaluation: (i) sensors position and velocity, and (ii) sensors position, velocity, and Euler angles.

Table 2 collects the conditions applied in each test. Main output of all the tests was the detection of a healthy or pathological pattern, and for the latter the further identification of the damage location, i.e., left or right hemisphere (LHD or RHD, respectively), for a total of three prediction classes. The additional output of the left (L) and right (R) distinction was also evaluated in four tests, increasing to six the prediction classes for this kind of tests.

Tests were performed on the complete dataset of all the subjects and the prediction accuracy of both the LDA and RF algorithms was evaluated according to Nuzzi et al. [34,35]. For the assessment of the RF algorithm accuracy, the OOB approach was also used. Results were averaged on 200 consecutive tests.

## 3. Results

Evaluating the OW according to the average window strategy, an OW length of 1/10 resulted equal to 0.27 s, and reached 0.33 s increasing the sample at 1/7 of the motion.

### 3.1. Intention Prediction

Table 3 collects mean value and standard deviation (SD) of LDA accuracy and RF OOB, on 200 tests performed considering the whole dataset of all the acquisitions, and the subsets of data gathered from healthy subjects and pathological patients. Results of tests performed without distinction of left and right limb are reported in bold font.

**Figure 2:**
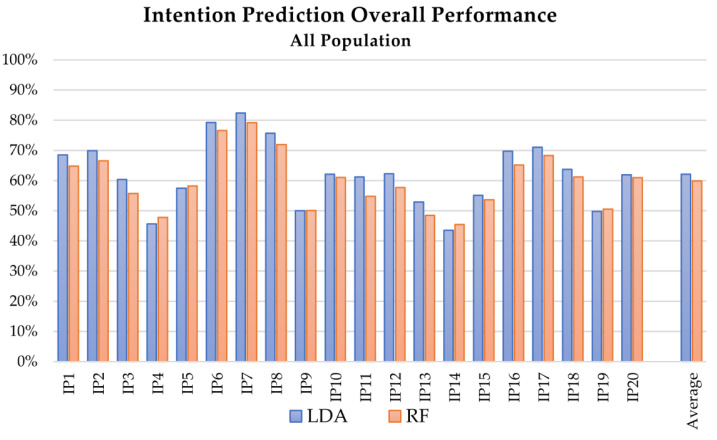
Mean accuracy of the intention prediction for the analysis of complete dataset (healthy and pathological subjects) with respect to the applied MLT: blue and red the results of the testing with LDA and RF, respectively.

**Figure 3:**
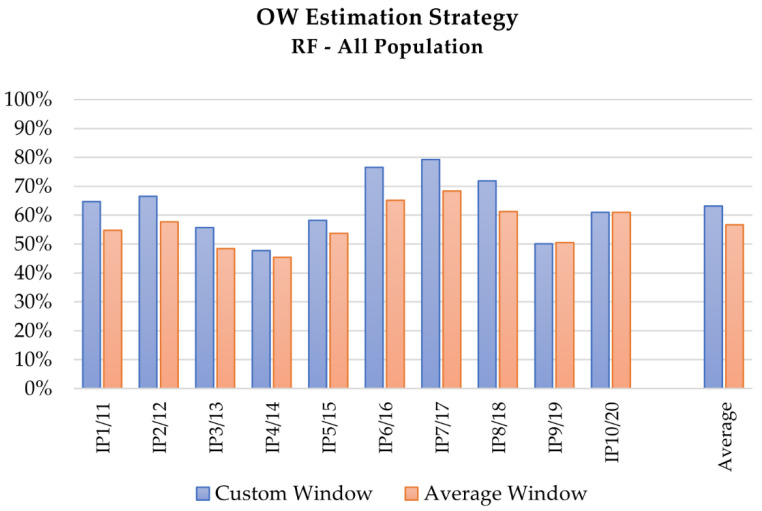
Mean accuracy of the intention prediction for the analysis of complete dataset (healthy and pathological subjects) with the RF algorithm, with respect to the OW estimation strategy: blue and red the results of the testing with custom and average window, respectively.

**Figure 4:**
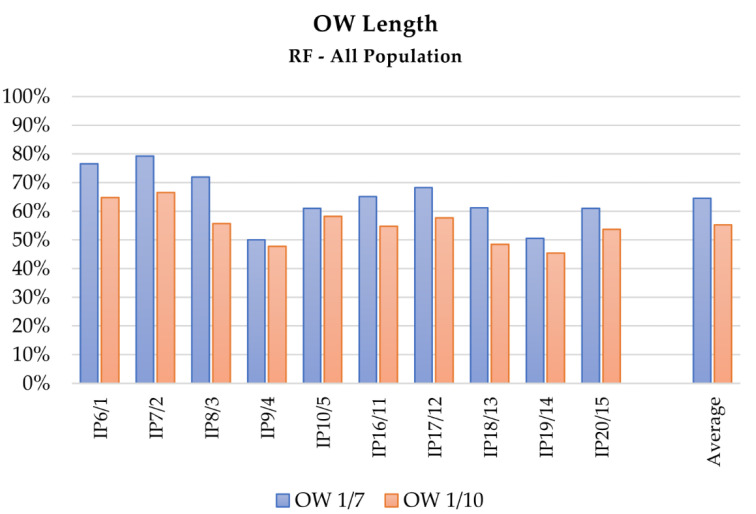
Mean accuracy of the intention prediction for the analysis of complete dataset (healthy and pathological subjects) with the RF algorithm, with respect to the OW length: blue and red the results of the testing with 1/7 and 1/10 of the motion time length, respectively.

**Figure 5:**
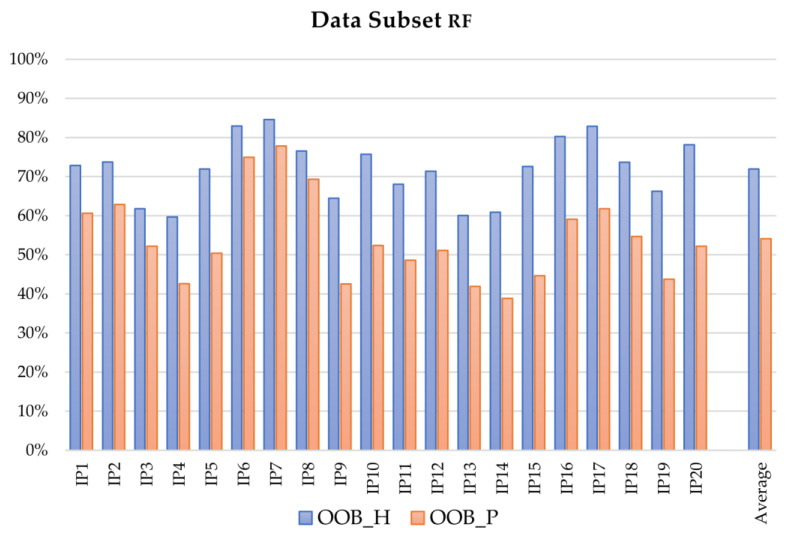
Mean OOB accuracy of the intention prediction for the analysis of healthy and pathological subsets with the RF algorithm: blue and red the results of the testing referring to healthy and pathological populations, respectively.

The overall performance comparison of LDA and RF algorithms in the intention prediction is presented at a glance in Figure 2, Figure 3 and Figure 4, which depict the results obtained with the RF algorithm for different OW estimation strategies and lengths respectively, whereas Figure 5 describes the mean computed accuracy of RF with respect to the evaluated data subset.

The RF algorithm demands an average training time of 1.14 s (range: 0.87–1.88), which decreases to an average value of 0.078 s (range: 0.035–0.28) for LDA. The prediction time was computed only for the tests with higher accuracy, i.e., IP1, IP2, IP6, and IP7. The average prediction time was 31 × 10^−4^ s (range: 30 × 10^−4^–33 × 10^−4^) for RF and 11 × 10^−5^ s (range: 10 × 10^−5^–12 × 10^−5^) for LDA.

### 3.2. Health Condition Detection

Table 4 depicts mean value and SD of LDA and RF accuracy, and of RF OOB, on 200 tests performed considering the whole dataset of all the acquisitions, composed by the data gathered from both healthy subjects and pathological patients. Results of tests performed without distinction of left and right limb are reported in bold font.

**Figure 6:**
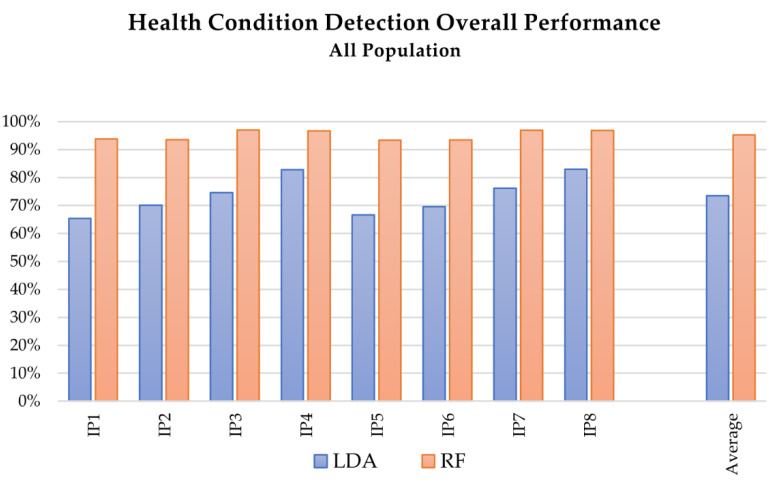
Mean accuracy of the health condition detection for the analysis of complete dataset (healthy and pathological subjects) with respect to the applied MLT: blue and red the results of the testing with LDA and RF, respectively.

**Figure 7:**
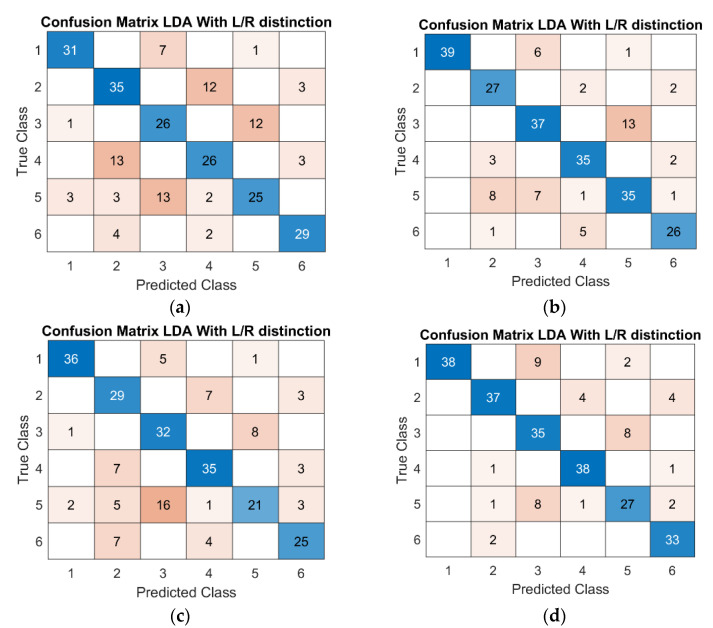
Example of LDA confusion matrixes with left and right limb distinction for (**a**) HD2, (**b**) HD4, (**c**) HD6, and (**d**) HD8. Testing dataset is 15% of the original data.

**Figure 8:**
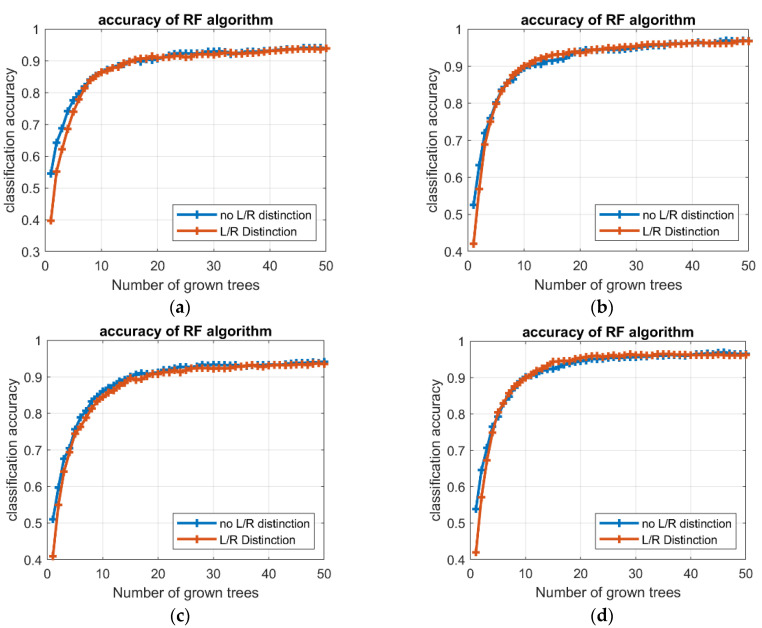
Example of RF accuracy with respect to the number of trees, for (**a**) HD1 (in blue) and HD2 (in red), (**b**) HD3 (in blue) and HD4 (in red), (**c**) HD5 (in blue) and HD6 (in red), and (**d**) HD7 (in blue) and HD8 (in red).

Figure 6 depicts at a glance the overall performance comparison of LDA and RF algorithms in the health condition detection. Figure 7 collects some examples of LDA confusion matrixes for tests including left and right limb distinction, whereas Figure 8 presents examples of the trend of RF accuracy with respect to the number of trees, for different tests.

Average training time for the RF algorithm was 1.56 s (range: 0.24–0.32), which decreased to an average value of 0.057 s (range: 0.031–0.072) for LDA. The average prediction time was 11 × 10^−2^ s (range 9 × 10^−2^–13 × 10^−2^) for RF and 9 × 10^−3^ s (range 6 × 10^−3^–11 × 10^−3^) for LDA.

## 4. Discussion

From a methodological perspective, the assessment of the observation window (OW) according to the custom window strategy should be preferred to the average window approach, because it grants a personalized solution which is flexible to subject- and trial-dependent peculiarities. Besides, the average window strategy offers a more robust approach, grounding on a dataset that, if properly populated, can provide probabilistically significant values and statistically sound indications for the OW definition. Nevertheless, in practical applications the time length of a naturally performed movement is typically an unknown quantity, since it cannot be foresight before the actual execution of the movement itself. According to these considerations, the average window approach results particularly suitable for integrations in systems requiring real-time dynamics, such as human–robot collaborations in working environment, whereas the custom window strategy better suits those applications demanding for high accuracy and results customization over timing, such as diagnostic evaluations in clinical environment.

The dualism of this issue reflects in the dual approach adopted for the analysis of the reaching task and of the current dataset. In fact, the prediction of the subject’s intention of moving towards a target among a set of possible choices can be easily contextualized in the industrial daily practice. For instance, in assembly operations a cobot could ease the task execution by foreseeing the worker’s intention of performing an action and approaching or moving the necessary components consequently. The health condition detection on the contrary could support the physician in discriminating the potential pathologies that afflict the subject or in quantitatively assessing the state of the rehabilitation process.

Comparing the overall performances of linear discriminant analysis (LDA) and random forest (RF) algorithms (see Figure 2 and Figure 6), LDA allows slightly better results to be reached for the intention prediction. Considering the whole dataset, the accuracy of RF was 79.22% at best (test IP_7), compared to 82.42% for LDA, whereas the average accuracy results were 59.91% for RF and 62.19% for LDA. On the contrary, RF proved to be particularly suitable for health condition detection, allowing accuracies of over 90% in all the tests.

Focusing on the contribution of the OW evaluation strategy in the performance of LDA and RF as intention predictors, the results collected in Table 3 emphasize that tests performed adopting the custom window method, an OW of 1/7, and considering the whole dataset of healthy and pathological subjects obtained better results, as Figure 3, Figure 4 and Figure 5 depict at a glance. This behavior can be expected if considering that a fixed OW does not allow compensating for possible intra-subjects or -trials velocity variations, and cannot assure the achievement of a minimum amount of travelled space within the analyzed portion of signal. This aspect can be particularly relevant for the analysis of pathological subjects, since their affected movements often induce a slower motion. As a consequence, a feature based on time hinders the potentiality of the method, whereas features based on spatial criteria could in this sense provide more information. Coherently, tests performed with longer portions of signals, i.e., 1/7 of the actual motion length, provide better results. Although the wider the window, the better the obtained result, a proper maximum length limit should be imposed for the OW length definition, since the primary aim of the analysis is a prediction of the motion evolution, whereas too wide OWs would translate into a classification of the movements instead.

In the health condition detection, LDA results less sensitive to variations in the OW length. As Table 4 describes, differences between the mean accuracies (around 1%) are comparable with the SD value.

According to these considerations, relevant differences in the performance of both LDA and RF can be detected when comparing tests evaluating the complete dataset and tests performed on the subsets of pathological and healthy subjects. For example, RF algorithm presents differences close to 4% and 11% with the two subsets, respectively. The difference with the subset of pathological subjects decreases for tests that use only acceleration as features. The LDA algorithm, as reported in Table 3, presented similar results.

Also considering the performance of the applied MLT as intention predictors with respect to the evaluated features, the OW length had a decisive influence on the final accuracy of both LDA and RF algorithms in tests using features extracted from Euler angles, sensor position, and velocity: The accuracy improved at least 10% in tests with OW length set at 1/7. The improvement decreased at about 5% (SD close to 1%) when acceleration-related features were included. This behavior could be partially justified if considering that acceleration signals are not measured but computed by double numerical derivation, and this process introduces noise in the signal, influencing the performance. As Figure 2 depicts, the tests including acceleration-related features (IP4, IP5, IP9, IP10, IP14, IP15, IP19, IP20) presented lower accuracies than the ones without their contribution; better results were obtained when the number of classes was lower, i.e., when the distinction between right and left limb performing the reaching was neglected.

For both LDA and RF, the addition of features computed with data derived from the sensors of shoulder and trunk does not significantly affect the obtained accuracy. In fact, the primary role of the trunk sensor was to validate the hypothesis that the elastic band used to fix the trunk to the chair worked as effective constraint, preventing the subject from unintentional movements. For the shoulder sensor instead, the introduced variation of performances considering the additional features (around 1%) was comparable with the SD amplitude and cannot be distinguished from to the results variability due to the random extraction of samples for the creation of the training and testing datasets. Despite this, the computational burden increased.

The set of included features also affected the results of the health condition detection. As Table 4 describes, better results were achieved including the Euler angle-related features, i.e., tests HD3, HD4, HD7, and HD8. The best results were obtained with RF in test HD3 (see Figure 6), in which features extracted from Euler angles were included and a wider OW was considered (OOB accuracy of 97.00% SD 0.0039, and AVG accuracy of 98.21% SD 3.38 × 10^−1^^6^). For the LDA algorithm, the accuracy ranged from 65% of HD1, presenting 3 classes (healthy, LHD, RHD), to 83% of HD8, which reached 6 classes discriminating also between the left and right limb. As the confusion matrixes in Figure 7 depict, regardless of the inclusion of additional classes, the algorithm still preserved its discrimination performance.

Finally, the time factor should be analyzed. Focusing on the training phase, the average time required to train the RF algorithm was considerably higher than LDA in both intention prediction (3.94 and 0.09 s, respectively) and health condition detection (1.5 and 0.05 s, respectively). Nevertheless, the training time for RF was related to the number of trees in the forest, and higher accuracies could be achieved with wider forest. Besides, the improvement in the accuracy tended to reduce as the number of trees increases, i.e., the accuracy profile converges toward an asymptotic condition; a set of preliminary analyses identified in 40 and 20 trees acceptable limits for the intention prediction and health condition detection, respectively (see Figure 8). For the testing phase, the LDA algorithm revealed shorter times than RF, for both intention prediction and health condition detection; the mean values of estimation time were 3.42 × 10^−4^ and 4.65 × 10^−3^ s for LDA and RF, respectively, in prediction, and decreased to 3.62 × 10^−5^ for LDA and 6.67 × 10^−4^ s for RF, in detection.

Although the analyzed dataset included a remarkable amount of data, further acquisition campaigns could improve the quality of the results. For instance, the data sample could be enlarged by subjects’ age and pathological conditions (such as similar elapsed time from the stroke event), allowing for stratified analyses, or improved in quality, e.g., better balancing the presence of right- and left-handed subjects, allowing for functional comparisons. Besides, further data acquisition campaigns could focus on the experimental setup, for instance including new sensors. Adding accelerometers and/or IMU inertial sensors would assure to gather actual acceleration data, allowing an experimental comparison with the results of the tests carried out with the acceleration features. Finally, to better understand the goodness of the models, further data analyses could also estimate indexes useful for the identification of type II errors, such as F1-score or G-index.

## 5. Conclusions

In this paper the human reaching movement has been analyzed comparing the performance of linear discriminant analysis (LDA) and random forest (RF) in: (i) predicting the subject’s intention of moving toward a specific direction or target (*intention prediction*), and (ii) discriminating if the subject is performing the reaching according to a healthy or pathological pattern, and in this case, the damage location (*health condition detection*). An experimental campaign of 31 subjects was designed and performed, and data acquired with wearable electromagnetic sensors were analyzed with LDA and RF machine learning techniques (MLT). Several tests with different configurations of observation window (OW) evaluation strategies and length, features, and data subsets were carried out.

According to the results obtained evaluating the current dataset, intention prediction is more sensitive than health condition detection to variations in the OW length. In conclusion, although both the MLT demonstrated a good accuracy, LDA revealed better results in terms of accuracy, training time, and prediction time for the intention prediction, whereas RF proved to be particularly suitable for health condition detection.

## Figures and Tables

**Table 1:** Synthesis of the applied conditions in the tests performed for the intention prediction. In the table, source signals are described as sensor position (SP), sensor velocity (SV), sensor acceleration (SA), and sensor Euler angles (SEA); L/R indicates the left and right limb distinction.

	Data Setup				Features				Additional Output
	OW Evaluation		OW Lengths		Source Signal				L/R Distinction
Test	Custom Window	Average Window	1/7	1/10	SP	SV	SA	SEA	
IP1	x			x	x	x			x
IP2	x			x	x	x		x	x
IP3	x			x	x				x
IP4	x			x			x		x
IP5	x			x			x		
IP6	x		x		x	x			x
IP7	x		x		x	x		x	x
IP8	x		x		x				x
IP9	x		x				x		x
IP10	x		x				x		
IP11		x		x	x	x			x
IP12		x		x	x	x		x	x
IP13		x		x	x				x
IP14		x		x			x		x
IP15		x		x			x		
IP16		x	x		x	x			x
IP17		x	x		x	x		x	x
IP18		x	x		x				x
IP19		x	x				x		x
IP20		x	x				x		

**Table 2:** Synthesis of the applied conditions in the tests performed for the health condition detection. For all tests, the average window approach was adopted as OW evaluation strategy. Source signals are described as sensor position (SP), sensor velocity (SV), sensor acceleration (SA), and sensor Euler angles (SEA); L/R indicates the left and right limb distinction.

	Data Setup		Features				Additional Output
	OW Lengths		Source Signal				L/R Distinction
Test	1/7	1/10	SP	SV	SA	SEA	
HD1	x		x	x			
HD2	x		x	x			x
HD3	x		x	x		x	
HD4	x		x	x		x	x
HD5		x	x	x			
HD6		x	x	x			x
HD7		x	x	x		x	
HD8		x	x	x		x	x

**Table 3:** Results of the performed tests on the complete dataset and the subsets of data acquired by heathy subjects and pathological patients. For each data sample, mean and SD of accuracy A and OOB are presented for LDA and RF, respectively. In bold font the data referring to tests without distinction of left and right limb.

	All Population				Healthy Subjects				Pathological Subjects			
	LDA		RF		LDA		RF		LDA		RF	
Test	A_mean_	A_SD_	OOB_mean_	OOB_SD_	A_mean_	A_SD_	OOB_mean_	OOB_SD_	A_mean_	A_SD_	OOB_mean_	OOB_SD_
IP1	68.50%	0.028	64.75%	0.0077	81.00%	0.042	72.85%	0.032	64.52%	0.037	60.65%	0.010
IP2	69.89%	0.030	66.51%	0.0090	86.13%	0.036	73.73%	0.015	66.87%	0.031	62.85%	0.011
IP3	60.35%	0.029	55.72%	0.0086	73.11%	0.045	61.78%	0.016	56.59%	0.40	52.18%	0.012
IP4	45.65%	0.027	47.77%	0.0086	58.27%	0.054	59.71%	0.015	41.71%	0.037	42.59%	0.011
**IP5**	**57.47%**	**0.030**	**58.26%**	**0.0078**	**73.56%**	**0.047**	**71.97%**	**0.013**	**52.09%**	**0.034**	**50.44%**	**0.010**
IP6	79.29%	0.024	76.56%	0.0069	88.97%	0.032	82.92%	0.011	77.55%	0.031	74.97%	0.0087
IP7	82.42%	0.022	79.22%	0.0068	92.80%	0.027	84.60%	0.010	81.01%	0.028	77.81%	0.0084
IP8	75.70%	0.026	71.93%	0.0072	85.45%	0.041	76.58%	0.013	71.57%	0.032	69.32%	0.0097
IP9	50.03%	0.031	50.08%	0.0082	63.84%	0.054	64.48%	0.016	45.02%	0.038	42.56%	0.011
**IP10**	**62.14%**	**0.027**	**61.02%**	**0.0078**	**78.82%**	**0.045**	**75.70%**	**0.012**	**55.17%**	**0.037**	**52.38%**	**0.010**
IP11	61.20%	0.029	54.79%	0.0084	78.80%	0.041	68.04%	0.015	55.03%	0.038	48.61%	0.011
IP12	62.29%	0.029	57.68%	0.0086	83.26%	0.042	71.40%	0.015	56.54%	0.036	51.10%	0.011
IP13	52.92%	0.029	48.48%	0.0092	69.56%	0.049	60.05%	0.016	46.96%	0.035	41.90%	0.011
IP14	43.48%	0.028	45.42%	0.0090	59.57%	0.054	60.92%	0.015	37.48%	0.035	38.87%	0.010
**IP15**	**55.11%**	**0.029**	**53.69%**	**0.0082**	**74.09%**	**0.048**	**72.57%**	**0.013**	**45.47%**	**0.036**	**44.63%**	**0.010**
IP16	69.73%	0.026	65.16%	0.0085	86.80%	0.040	80.27%	0.012	64.28%	0.037	59.12%	0.011
IP17	71.04%	0.027	68.31%	0.0081	90.61%	0.034	82.88%	0.013	66.14%	0.033	61.81%	0.0098
IP18	63.68%	0.026	61.21%	0.0083	81.15%	0.044	73.65%	0.012	58.71%	0.036	54.72%	0.011
IP19	49.75%	0.029	50.56%	0.0089	70.04%	0.048	66.26%	0.014	44.11%	0.036	43.74%	0.011
**IP20**	**61.87%**	**0.027**	**61.00%**	**0.0086**	**83.59%**	**0.040**	**78.17%**	**0.011**	**53.92%**	**0.039**	**52.22%**	**0.010**

**Table 4:** Results of the performed tests on the complete dataset of data acquired by heathy subjects and pathological patients. For each data sample, mean and SD of accuracy A are presented for LDA and RF, and of OOB for RF. In bold font the data referring to tests without distinction of left and right limb.

	LDA		RF			
Tests	A_mean_	A_SD_	OOB_mean_	OOB_SD_	A_mean_	A_SD_ (·10^−16^)
**HD1**	**65.39%**	**0.026**	**93.80%**	**0.0047**	**95.83%**	**4.51**
HD2	70.11%	0.029	93.57%	0.005	94.05%	4.51
**HD3**	**74.59%**	**0.026**	**97.00%**	**0.0039**	**98.21%**	**3.38**
HD4	82.78%	0.023	96.72%	0.0036	97.62%	7.90
**HD5**	**66.63%**	**0.027**	**93.40%**	**0.0051**	**94.05%**	**4.51**
HD6	69.57%	0.030	93.50%	0.0052	91.07%	2.26
**HD7**	**76.22%**	**0.025**	**96.96%**	**0.0037**	**99.40%**	**4.51**
HD8	83.03%	0.022	96.86%	0.0033	98.21%	3.39

## Data Availability

Not applicable.
